# Herpes zoster is not associated with subsequent gastrointestinal cancer: data from over 200,000 outpatients in Germany

**DOI:** 10.1007/s00432-023-05432-4

**Published:** 2023-09-27

**Authors:** Catherine Leyh, Christoph Roderburg, Tom Luedde, Sven H. Loosen, Karel Kostev

**Affiliations:** 1 Department of Gastroenterology, Hepatology and Infectious Diseases, Medical Faculty, University Hospital Düsseldorf, Heinrich Heine University Düsseldorf, Moorenstraße 5, 40225 Düsseldorf, Germany; 2 Center for Integrated Oncology, Aachen Bonn Cologne Düsseldorf (CIO ABCD), Düsseldorf, Germany; 3 IQVIA, Frankfurt, Germany

**Keywords:** Varicella zoster virus, Herpes zoster, Gastrointestinal cancer, Epidemiology, Malignancy

## Abstract

**Purpose:**

Gastrointestinal (GI) cancers are an increasing global health challenge. Viral diseases play an important role in the development of GI cancers. For example, Epstein-Barr virus, which belongs to the human herpesvirus family, is a well-recognized risk factor for the development of gastric cancer. The purpose of this study was to investigate a possible association between varicella-zoster virus reactivation and subsequent diagnosis of GI cancer.

**Methods:**

In this retrospective cohort study, a total of 103,123 patients with a first diagnosis of herpes zoster (HZ) between 2005 and 2021 were propensity score matched to a cohort of 103,123 patients without HZ. Patient data was extracted from the Disease Analyzer database (IQVIA). The incidence of GI cancer was compared as a function of HZ. Cox regression analysis was used to examine the association between HZ and GI cancer.

**Results:**

Over a follow-up period of up to 10 years, the incidence of GI cancer did not differ between the two cohorts (HZ cohort 2.26 cases per 1000 patient-years vs. non-HZ cohort 2.37 cases per 1000 patient-years, p = 0.548). In regression analysis, HZ was not associated with an increased risk of developing GI cancer (HR: 0.97; 95% CI 0.89–1.05). Furthermore, no significant effect of the presence of HZ on the incidence of different GI cancer entities was found.

**Conclusion:**

In this retrospective cohort study consisting of well-matched patients, we observed no significant association between a HZ infection and the development of GI cancer during a long-term follow-up.

## Introduction

Varicella-zoster virus (VZV) is a neurotropic virus that belongs to the human herpesvirus family. After primary infection, the virus persists in neural tissue for life (Patil et al. 2022). Accordingly, VZV can cause two types of disease: (i) Chickenpox, the initial exogenous infection, and (ii) herpes zoster (HZ), the clinical manifestation of an endogenous reactivation of persistent latent VZV from sensory ganglia (Cohen 2013). HZ is particularly common in the elderly. In addition, immunocompromised patients, such as those who have undergone organ transplantation, malignant disease or human immunodeficiency virus (HIV) infection, are particularly susceptible to HZ (Patil et al. 2022).

Gastrointestinal (GI) cancers account for approximately one quarter of all cancers worldwide and are responsible for 35% of all cancer-related deaths. With an aging general population, the incidence is expected to further increase over the next decades (Arnold et al. 2020).

An association between viruses and GI cancers is already well established for human papilloma virus (HPV) and Epstein-Barr virus (EPV), the latter of which also belongs to the human herpesvirus family. HPV is associated with the development of oropharyngeal and anal cancers, while EBV is associated with the development of stomach cancer (Mirzaei et al. 2018). Another well-studied association is the occurrence of hepatobiliary tumors due to hepatitis B or C virus infection (Galle et al. 2018).

Both diseases, HZ and GI cancer, are associated with immunosuppression and an older age. The occurrence of HZ as a complication in patients with overt cancer-disease is a well-established and studied association (Cohen 2013; Tayyar and Ho 2023). Recently, it has been hypothesized that the manifestation of an HZ may also be indicative for the presence of an occult tumor as the hereby caused immunosuppression may favor the manifestation of HZ (Cohen 2013). However, there is currently insufficient evidence for the opposite situation, i.e. tumor development following HZ infection as has been demonstrated for other members of the human herpesvirus family. The aim of this study was therefore to investigate the occurrence of GI cancer after HZ.

## Materials and methods

### Database

This retrospective cohort study is based on data from the Disease Analyzer database (IQVIA). This database, which has been used in several previous studies focusing on cancer (Schiffmann et al. 2020; Loosen et al. 2022; Jacob et al. 2023), contains anonymous health-record data comprising diagnoses, prescriptions, and basic medical and demographic data from computer systems used in the office based practices (Rathmann et al. 2018). The database covers approximately 3–5% of all office-based practices in Germany. The sampling method for the Disease Analyzer database uses statistics from the German Medical Association to determine the panel design according to specialty groups, federal states, community size category, and age of the physician. The panel of practices included in the Disease Analyzer database has previously been shown to be representative of general and specialist practices in Germany (Rathmann et al. 2018).

### Study population

**Fig. 1:**
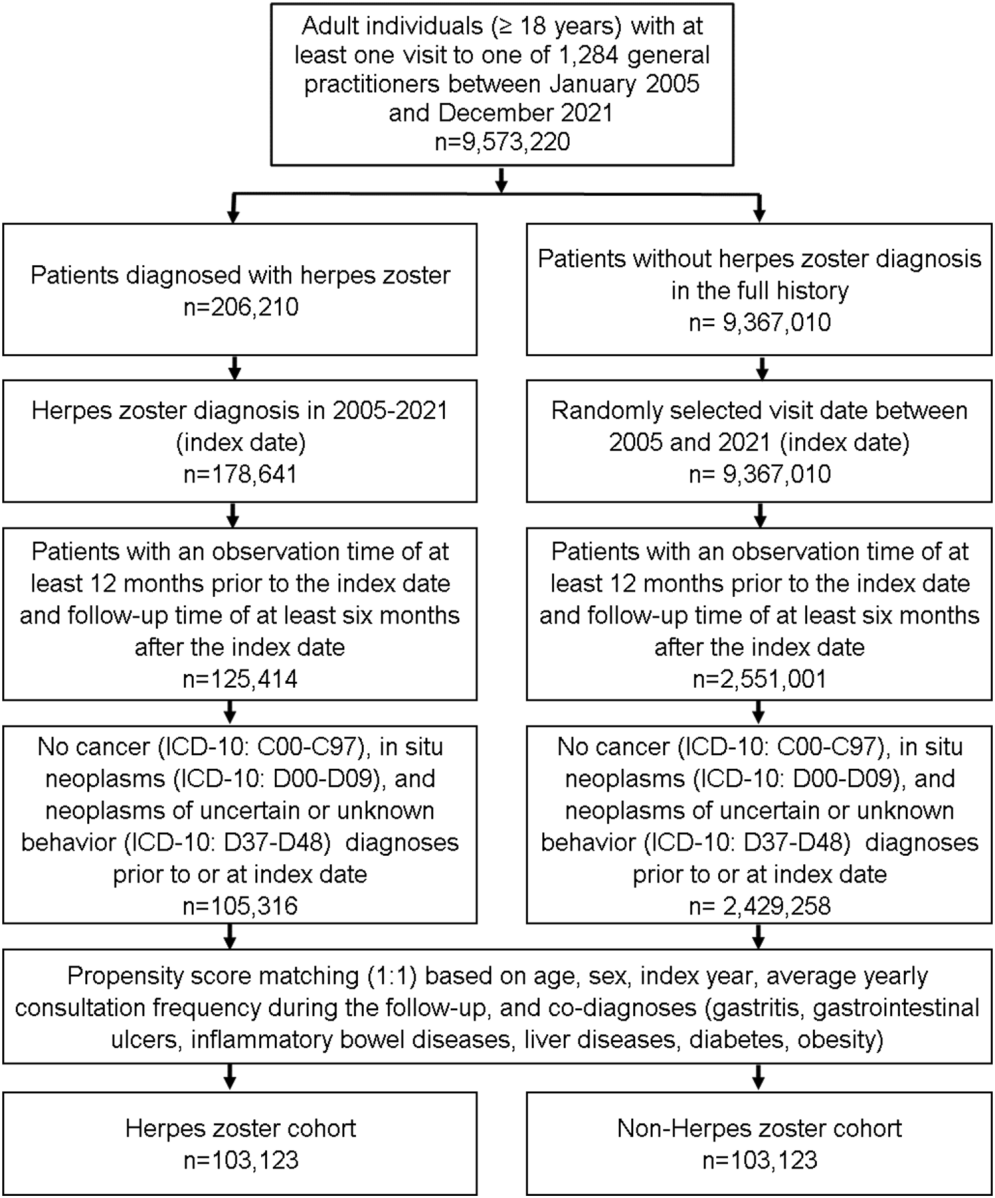
Selection of study patients

For the present study, we included patients aged ≥ 18 years with a first diagnosis of herpes zoster (HZ, ICD-10: B02) in 1284 general practices in Germany between January 2005 and December 2021 (index date; Fig. 1). The timepoint of the first diagnosis of HZ was set to be the index date. Further inclusion criteria were data-availability of at least 12 months before the index date and a follow-up period of at least six months after the index date. Patients with a diagnosis of cancer (ICD-10: C00-C97), in situ neoplasm (ICD-10: D00-D09), and neoplasm of uncertain or unknown behavior (ICD-10: D37-D48) before or at the index date were excluded. After applying similar inclusion criteria, individuals without a documented HZ diagnosis were matched to HZ patients using nearest neighbor propensity score matching (1:1) based on age, sex, index year, average annual consultation frequency during the follow-up, and co-diagnoses (gastrointestinal ulcers (ICD-10: K25-K28), gastritis (ICD-10: K29), inflammatory bowel diseases (ICD-10: K50, K51), liver diseases (B18, K70-K77), diabetes (ICD-10: E10-E14), and obesity (ICD-10: E66)). For the non-HZ cohort, the index date was that of a randomly selected visit between January 2005 and December 2021. The selection of study patients is painted out in Fig. 1.

### Study outcomes and statistical analyses

The study outcomes were first-documented diagnoses of gastrointestinal cancers (GI cancer, ICD-10: C15-C26) in total as well as separately for colorectal (ICD-10: C16, C18), stomach (ICD-10: C20), pancreas (ICD-10: C25) and liver (ICD-10: C22) cancers over a maximum follow-up period of 10 years after the index date. Baseline characteristics between the HZ and non-HZ cohort were compared using the Wilcoxon signed-rank test for continuous variables, the McNemar test for categorical variables with two categories, and the Stuart-Maxwell test for categorical variables with more than two categories. The incidence of GI cancer in the HZ and non-HZ cohort was estimated as number of cases per 1000 patient-years and displayed as bar graphs. Differences in the incidence of GI cancer between both groups were assessed using the Wilcoxon rank test. Univariable Cox regression analysis was performed to investigate the association between HZ and GI cancer. The results of the Cox regression model are shown as hazard ratios (HR) and 95% confidence intervals (CI). Cox regression analyses were performed separately for males, females and four age groups. Due to multiple comparisons and a large number of patients, a p value of < 0.01 was considered statistically significant. Analyses were performed using SAS version 9.4 (SAS Institute, Cary, USA).

## Results

### Baseline characteristics of the study sample

The present study included 103,123 individuals with HZ and 103,123 individuals without HZ. The baseline characteristics of the study population are summarized in Table 1. The mean age was 58.8 years and 61.2% were female. Due to matching, no significant differences in age, sex, visit frequency and comorbidities were observed between the two cohorts (Table 1). Patients visited their general practitioner an average of 7.8 times per year during follow-up. Within 12 months before the index date, 20.1% of patients were diagnosed with gastrointestinal ulcers and 23.4% with gastritis. A diagnosis of liver disease was documented in 10.9% of patients during this period, and 17.3% of patients had diabetes mellitus. Inflammatory bowel disease was documented in less than 1% of patients.

**Table 1:** Baseline characteristics of the study sample (after propensity score matching)

Variable	With herpes zoster (%) N = 103,123	Without herpes zoster (%) N = 103,123	p value
Age (mean, SD)	58.8 (17.5)	58.8 (17.6)	0.684
Age ≤ 50	30.1	30.2	0.781
Age 51–60	20.2	20.3	
Age 61–70	20.4	20.3	
Age > 70	29.3	29.2	
Women	61.2	61.2	1.000
Men	38.8	38.8	
Number of physician visits per year during the follow-up (mean, SD)	7.8 (3.9)	7.8 (3.9)	1.000
Diagnoses documented within 12 months prior to or at index date			
Gastrointestinal ulcers	20.1	20.1	1.000
Gastritis	23.4	23.4	1.000
Inflammatory bowel diseases	0.9	0.9	1.000
Liver diseases	10.9	10.9	1.000
Diabetes	17.3	17.3	1.000
Obesity	10.7	10.7	1.000

Proportions of patients given in %, unless otherwise indicated

*SD* standard deviation

### Association between herpes zoster and gastrointestinal cancer

**Fig. 2:**
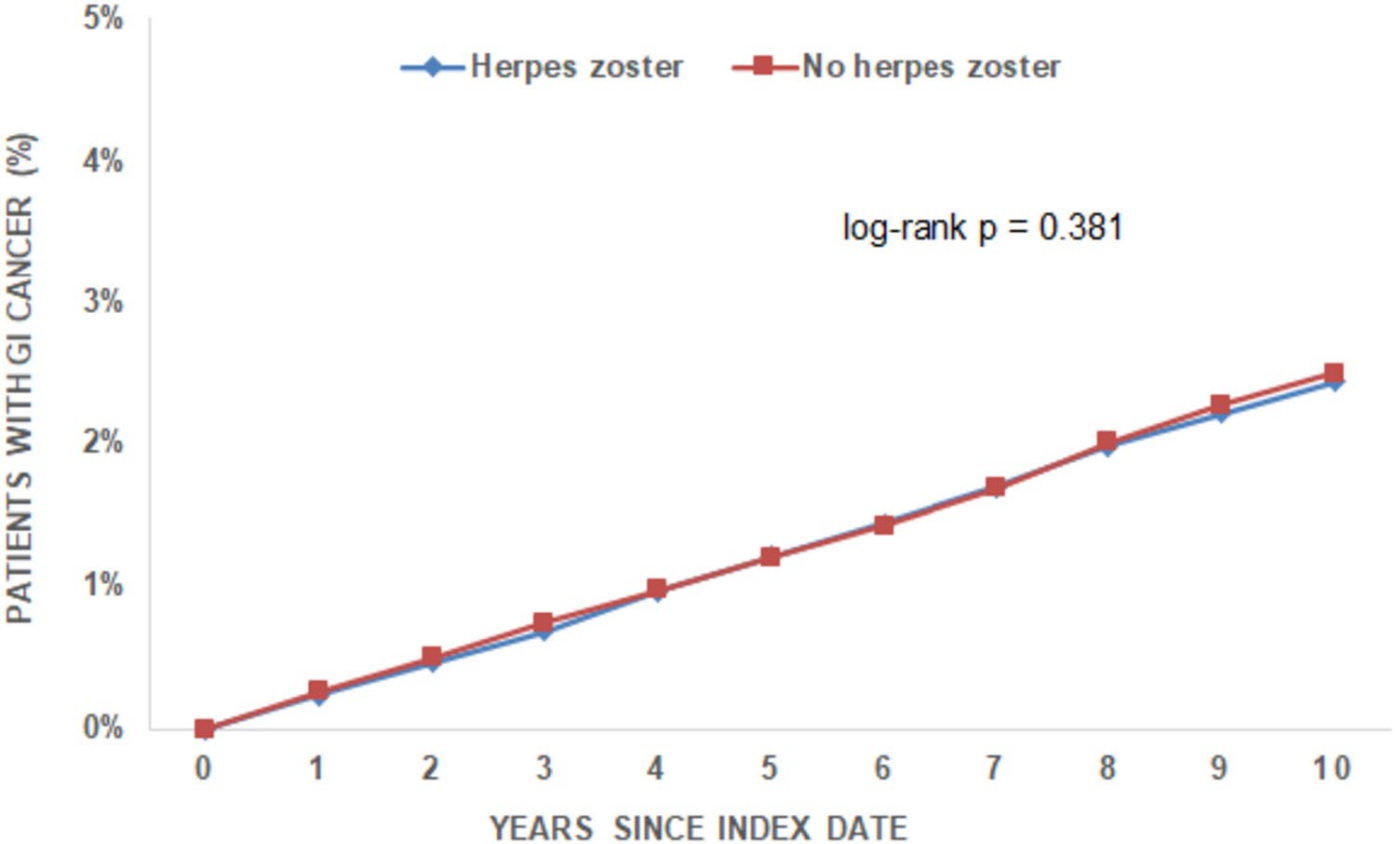
Cumulative incidence of GI cancer among individuals with and without herpes zoster depending on the years after HZ diagnosis

**Fig. 3:**
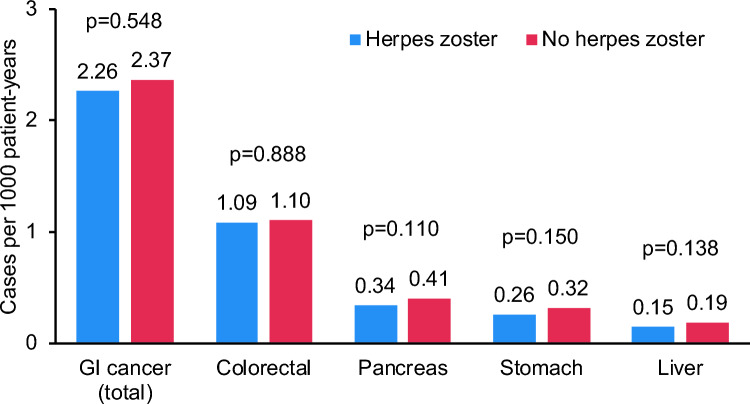
Cumulative incidence of GI cancers (cases per 1000 patient-years) in individuals with and without herpes zoster

There was no significant difference in the total number of all GI cancers between the two cohorts (HZ cohort vs. non-HZ cohort). The incidence of GI cancer was 2.26 cases per 1000 patient-years in the HZ cohort and 2.37 cases per 1000 patient-years in the non-HZ cohort (p = 0.548) during up to 10 years of follow-up. Of note, the incidence did not differin the first years after HZ diagnosis compared to patients without any HZ diagnosis (Fig. 2). Regarding the different GI tumor entities, no significant differences were observed between the incidence of colorectal, pancreatic, gastric and liver cancer in both cohorts (Fig. 3). Regression analysis showed no significant association between the presence of HZ and GI cancer (HR: 0.97; 95% CI 0.89–1.05). No significant association was found for the different tumor entities (colorectal cancer: HR: 1.01; 95% CI 0.90–1.13, gastric cancer: HR: 0.85; 95% CI 0.6–1.06, pancreatic cancer: HR: 0.84; 95% CI 0.69–1.03 and liver cancer: HR: 0.73; 95% CI 0.55–0.98). Furthermore, no significant association was observed in age- and sex-stratified analyses (Table 2).

**Table 2:** Association between herpes zoster and subsequent GI cancers in patients followed in general practices in Germany (univariable Cox regression models)

	HR (95% CI)	p value
Any GI cancer		
Total	0.97 (0.89–1.05)	0.380
Age ≤ 50	1.37 (0.99–1.89)	0.056
Age 51–60	0.94 (0.77–1.14)	0.509
Age 61–70	0.88 (0.76–1.03)	0.115
Age > 70	0.94 (0.84–1.05)	0.272
Women	0.98 (0.88–1.09)	0.671
Men	0.95 (0.85–1.07)	0.414
Colorectal cancer		
Total	1.01 (0.90–1.13)	0.823
Age ≤ 50	1.25 (0.79–1.97)	0.347
Age 51–60	1.02 (0.75–1.39)	0.888
Age 61–70	0.89 (0.71–1.12)	0.306
Age > 70	1.00 (0.85–1.18)	0.999
Women	0.98 (0.84–1.15)	0.808
Men	1.05 (0.88–1.25)	0.613
Stomach cancer		
Total	0.85 (0.68–1.06)	0.155
Age ≤ 50	1.36 (0.62–2.98)	0.446
Age 51–60	0.65 (0.39–1.10)	0.107
Age 61–70	0.88 (0.58–1.33)	0.544
Age > 70	0.83 (0.59–1.16)	0.269
Women	0.85 (0.62–1.17)	0.318
Men	0.85 (0.62–1.17)	0.316
Pancreas cancer		
Total	0.84 (0.69–1.03)	0.084
Age ≤ 50	1.65 (0.62–4.40)	0.322
Age 51–60	0.69 (0.41–1.15)	0.151
Age 61–70	0.86 (0.58–1.27)	0.456
Age > 70	0.80 (0.61–1.04)	0.095
Women	0.85 (0.67–1.09)	0.210
Men	0.82 (0.59–1.13)	0.226
Liver cancer		
Total	0.73 (0.55–0.98)	0.039
Age ≤ 50	0.80 (0.11–5.72)	0.824
Age 51–60	0.82 (0.44–1.54)	0.543
Age 61–70	0.85 (0.47–1.53)	0.582
Age > 70	0.62 (0.41–0.94)	0.025
Women	0.86 (0.55–1.37)	0.535
Men	0.65 (0.45–0.96)	0.031

## Discussion

The fact that viruses of the human herpesvirus family can promote the development of malignancies has been demonstrated for EBV. EBV-patients have an increased risk of developing Hodgkin’s lymphoma in the follow-up of the infection (Shannon-Lowe et al. 2017; Roderburg et al. 2022). For gastrointestinal tumors in general a strong association with gastric cancer has been reported in several studies, including a large meta-analysis by Tavakoli et al. (Mirzaei et al. 2018; Tavakoli et al. 2020). Concerning Germany, however, only two small case–control studies were included in the aforementioned meta-analysis. A large cohort study based on data from the Disease Analyzer database (IQVIA) and involving 24,190 outpatients in Germany did not find a significant association between EBV and gastrointestinal tumors (Roderburg et al. 2022). However, the authors didn’t distinguish between the different GI tumor entities, so a possible significant association with gastric cancer cannot be ruled out. For colorectal cancer, the data are inconclusive regarding a possible association with EBV (Bedri et al. 2019). In contrast, in an Asian collective, EBV-associated intrahepatic cholangiocarcinoma has been described as a separate subgroup (Huang et al. 2021). However, it remains unclear whether other members of the herpesvirus family, in particular VZV, influence tumor development as it was first suggested in 1955 (Wyburn-Mason 1955).

By using a large database of more than 200,000 outpatients in Germany, we conducted a retrospective analysis to investigate the association between the occurrence of GI cancer after a diagnosis of HZ. Strikingly, in contrast to data suggesting increased cancer rates after HZ (Buntinx et al. 2005; Chiu et al. 2013; Cotton et al. 2013), our results did not identify a positive association between HZ and subsequent GI cancer after HZ during a 10-year follow-up period in a very large cohort of well-matched patients.

Both HZ and GI cancers are closely associated with immune dysfunction. Therefore, it has often been hypothesized that HZ may be associated with an occult tumor that is not yet clinically apparent but is already affecting the immune system (Patil et al. 2022). Although recent studies investigating this relationship have reported a significant association, the data remain largely inconclusive, particularly with regard to GI tumors (Buntinx et al. 2005; Schmidt et al. 2017; Sim et al. 2021). In a retrospective study published in 2005, Buntinx et al. described an increased risk of overall tumor development in women older than 65 years after an initial diagnosis of HZ. In an exploratory subgroup analysis, the authors further demonstrated an HR for the development of colorectal cancer of 4.001 (95% CI 1.123–14.257). However, only 1211 patients with HZ were included in this study, of which only 25 patients developed tumor disease during follow-up, limiting the statistical power of the results (Buntinx et al. 2005). Chiu et al. performed a retrospective study including a total of 38,743 patients with HZ and 116,229 patients without HZ. The risk of developing any tumor disease was increased in the first 2 years after diagnosis of HZ. Notably, after a total follow-up period of 5 years, there was no significant difference compared to the control group (Chiu et al. 2013). Cotton et al. (2013) also assessed the risk of developing tumor disease after HZ and showed that the risk was increased, especially in the first year after HZ diagnosis. Finally, Iglar et al. (2013) also showed that the association between HZ and tumor development is particularly strong within the first 6 months after diagnosis of HZ and tends to decrease over time. Therefore, one might speculate that the diagnosis of tumor disease within a short period after HZ might be indicative of a pre-existing but occult and undiagnosed tumor at the timepoint of HZ. In fact, HZ may be a symptom of tumor-associated immunodeficiency that favors VZV reactivation in these cases (Kim et al. 2021). In our study, we also assessed the incidence of GI cancer at different timepoints after HZ diagnosis. In contrast to the findings reported by Cotton et al. and Chiu et al. we did not find increased rates of GI cancer in the first years after HZ diagnosis. Therefore, regarding our data we cannot support the theory of HZ being indicative of occult GI cancer disease that might be more likely diagnosed after HZ-diagnosis.

Interestingly, the clinical course of HZ also seems to be relevant for the association with tumor development. Post-herpetic neuralgia (PHN) is an important complication of HZ that is particularly challenging to treat and occurs preferentially in immunocompromised patients. A study in an Asian cohort found out, that the occurrence of PNH was associated with subsequent tumor diagnosis, including the GI system, whereas patients with HZ but without PNH had a lower risk of tumor development (Sim et al. 2021).

To the best of our knowledge, we report for the first-time on the specific association of HZ and the subsequent GI cancer. In contrast to the aforementioned studies, in the present retrospective study, we focused on different GI cancer entities and examined also the long-term follow-up of the patients and did not identify a significant association between the two disease entities.

The main strength of our study is the large number of patients analyzed with a long follow-up time of up to 10 years. We performed sufficient matching of control patients without HZ, which further increased the quality of our data. However, the main limitations of our study are the retrospective study design and analyses based on ICD code data, lack of hospital data, mortality information, data on smoking status, alcohol use, physical activity, and detailed information on laboratory parameters, such as inflammation. Further, we did not review individual medical records in detail. It should be noted that we focused our analysis on the most common tumor entities of the GI tract and thus cannot exclude an association with less common tumor subtypes.

In conclusion, there was no association between HZ and subsequent GI-tumors. Our data therefore do not support the hypothesis, that VZV has pro-oncogenic effects during reactivation.

## Data Availability

The datasets used and/or analyzed during the current study are available from the corresponding author upon reasonable request.
